# Modeling of Scale-Dependent Bacterial Growth by Chemical Kinetics Approach

**DOI:** 10.1155/2014/820959

**Published:** 2014-07-03

**Authors:** Haydee Martínez, Joaquín Sánchez, José-Manuel Cruz, Guadalupe Ayala, Marco Rivera, Thomas Buhse

**Affiliations:** ^1^Facultad de Medicina, Universidad Autónoma del Estado de Morelos, Avenida Universidad 1001, 62209 Cuernavaca, MOR, Mexico; ^2^Centro de Investigaciones Químicas, Universidad Autónoma del Estado de Morelos, Avenida Universidad 1001, 62209 Cuernavaca, MOR, Mexico; ^3^Centro de Investigación sobre Enfermedades Infecciosas, INSP, 62100 Cuernavaca, MOR, Mexico; ^4^Facultad de Ciencias, Universidad Autónoma del Estado de Morelos, Avenida Universidad 1001, 62209 Cuernavaca, MOR, Mexico

## Abstract

We applied the so-called chemical kinetics approach to complex bacterial growth patterns that were dependent on the liquid-surface-area-to-volume ratio (SA/V) of the bacterial cultures. The kinetic modeling was based on current experimental knowledge in terms of autocatalytic bacterial growth, its inhibition by the metabolite CO_2_, and the relief of inhibition through the physical escape of the inhibitor. The model quantitatively reproduces kinetic data of SA/V-dependent bacterial growth and can discriminate between differences in the growth dynamics of enteropathogenic *E. coli*, *E. coli* *JM83*, and *Salmonella typhimurium* on one hand and *Vibrio cholerae* on the other hand. Furthermore, the data fitting procedures allowed predictions about the velocities of the involved key processes and the potential behavior in an open-flow bacterial chemostat, revealing an oscillatory approach to the stationary states.

## 1. Introduction

Kinetic curves deliver the dynamic fingerprint of time-evolving systems. This applies to the study of complex chemical systems [1], has been used to portray ecological population dynamics [2], and is perhaps true for bacterial systems despite their tremendous microscopic complexity. In this last sense, one may ask how much information about the underlying mechanism of bacterial growth dynamics could be extracted by evaluating bacterial growth curves. This might resemble the attempt to decipher the mechanism of a chemical system by studying its kinetic behavior.

In their attempt to predict bacterial growth kinetics in food substrates, Doona and coworkers [3–5] introduced the so-called chemical kinetics approach (CKA) to rationalize bacterial growth curves. Basically, the approach encompassed the treatment of bacteria, nutrients, and metabolites as chemical species involved in a number of coupled processes that obeyed the rules of chemical kinetics. The time-evolution of the various components was expressed by a set of coupled ordinary differential equations that were numerically resolved. Since bacterial growth is essentially nonlinear, the incorporation of positive or negative feedback allowed an integral, “all-in-one” description of evolving bacterial growth including the growth stages usually denominated as “lag,” “exponential,” “stationary,” and “death” phases [6–11] with just one model and without changing the parameters or their values. Hence the CKA relates to the exploration of the global and continuous bacterial growth dynamics and perhaps allows predictions as well as the identification and discrimination of complex mechanisms that could be at the origin of atypical growth behavior.

From the above viewpoint, the CKA is settled in between empirical laws and their extensions usually based on logistic curve fitting procedures [7–11] and the kinetic modeling at the biochemical level [12–14]. While the first approach allows only very limited insight into the mechanism behind bacterial growth dynamics, the second approach usually features a molecularly inclined view on selected bacterial processes but not on the overall growth dynamics. Thus the CKA is a mechanism-based simulation procedure that uses a coarse-grained dynamical viewpoint.

Peleg and Corradini [15] recently not only discussed the benefits of the CKA but also pointed out that the use of fixed order kinetics, as well as fixed stoichiometries in the CKA, could be a potential disadvantage in dealing with macroscopic and complex elements such as bacteria. This is readily understood by considering, for instance, the interaction between* one* bacterium and* one* nutrient molecule leading to some change in the bacteria, that is, the chemical equivalent of the conversion of the bacteria into a product. This would be the case of second-order kinetics. However, it is more likely that* one* bacterium interacts with* many* nutrient molecules to convert them into a “product.” This consideration can lead to a substantial increase in the formal kinetic order of such processes and because of the huge discrepancy in the particular orders, that is, very low order in the bacteria and very high order in the nutrient, they must not necessarily remain constant during the whole time-evolution. Hence a further evaluation of the CKA is pertinent so that it may be applicable to more complex growth patterns than treated so far.

**Figure 1:**
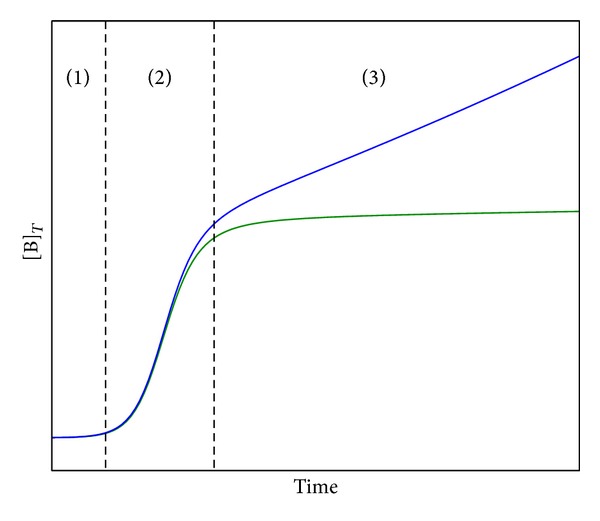
Sketch of typical SA/V-dependent EPEC growth curves ([B]_*T*_ = total bacteria concentration): (1) lag phase and (2) exponential growth independent of the SA/V and (3) later stage growth depending on the SA/V. If the SA/V increases also the rate of the later stage growth increases. Note that under common laboratory culturing conditions the SA/V remains relatively small so that the later stage growth may look stationary (green curve) which, however, does not appear to be the case at higher SA/V ratios (blue curve).

As one example of less usual bacterial growth patterns, among others [16, 17], we previously found that the rate of later stage bacterial growth of enteropathogenic* E. coli* (EPEC) was correlated to the liquid-surface-area-to-volume ratio (SA/V) of the own liquid bacterial cultures [18], that is, to the scaling properties of the bacterial culturing system in dispersion (Figure 1).

The SA/V effect was related to the generation of a volatile inhibitor metabolite, namely, CO_2_ [19], which was produced by the bacteria during and after their conversion into an anaerobic regime [20]. Such conversion occurs because of the progressive depletion of the dissolved oxygen in the medium. The rate by which CO_2_ escapes from the liquid cultures depends on the SA/V of the medium and its physical escape gives rise to a relief of inhibition.

Hence we reasoned that the relief of inhibition accounted for the SA/V-dependent growth rates. Moreover, equivalent to the curve shape of typical zero-order kinetics, the last growth stage of EPEC was characterized by a quasilinear increase of the bacterial concentration in time. The suggested scenario at the origin of such growth behavior was supported by complementary experiments as well as by a simplified kinetic model, which was built in the spirit of the CKA. Such kinetic CKA-inspired model reproduced qualitatively the growth curves in terms of an interplay between inhibition (generation of the inhibitor) and the relief of inhibition (escape of the inhibitor).

In this paper, we show that our former observations of SA/V-dependent EPEC growth as well as the present recordings of* E. coli JM83* and* Salmonella typhimurium* (also* Salmonella enterica* subsp.* enterica* ser. Typhimurium) growth can be* quantitatively* reproduced by the same model based on the CKA. This was in contrast to the* Vibrio cholerae* growth patterns that seemingly follow a distinct growth dynamics. Hence our modeling indicates not only that the SA/V-effect, as well as the particular kinetic curve shapes, is caused by a common mechanism for the different bacteria but also that changes may occur when the global growth dynamics differ.

To our knowledge, we demonstrate for the first time that the CKA combined with data fitting can discriminate between the growth dynamics of different bacteria simply by analyzing the shape of the kinetic growth curves. Moreover, the ranges of the extracted kinetic parameters allowed us to estimate the velocities of the key steps of bacterial autocatalytic growth, growth inhibition, and its relief by the escape of the inhibitor. The applied procedure also permitted predictions of the possible growth behavior in a bacterial chemostat, shedding more light on the dynamic properties of SA/V-dependent bacterial growth.

## 2. Material and Methods

Model calculations were performed with the simulation-adjustment software package* Sa3* written by D. Lavabre, Laboratoire des IMRCP, Université Paul Sabatier, Toulouse, France, that is freely available at http://cinet.chim.pagesperso-orange.fr/index.html#. The general algorithm for the numerical integration of the differential equations was based on a semi-implicit fourth-order Runge-Kutta method with stepwise control for stiff ordinary differential equations. The minimization algorithms for the adjustment of the kinetic parameter values were of either the Powell type or random walk simulated annealing. Fitted rate parameters were automatically and iteratively returned to the numerical integration until a minimum in the residual error was reached.

The bacterial strains EPEC,* E. coli JM83*,* Salmonella typhimurium*, and* Vibrio cholerae* were grown under the same standard procedures with fixed initial bacterial concentrations in nutrient-rich liquid media (batch cultures) as previously described [18, 19]. The optical density (OD) was measured in a UV-VIS spectrophotometer cuvette at 600 nm and at 37°C ± 0.3°C under gentle magnetic stirring. The scaling properties were established by varying the height of the liquid column in the spectrophotometric cuvette or by obstructing the liquid/gas surface area (SA/V = 0 cm^−1^) with a premolded paraffin block that tightly fitted the inside of the cuvette. The total bacterial concentration was assumed directly proportional to the OD.

## 3. Results and Discussion

### 3.1. Model Description

**Scheme 1:**
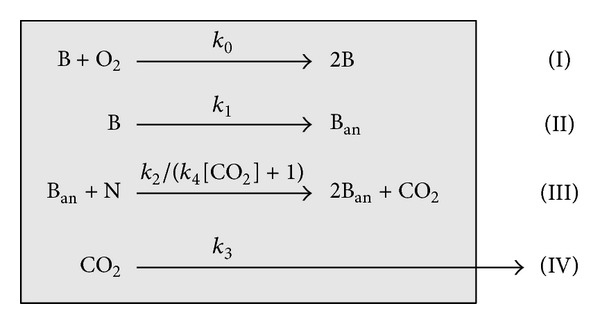
Kinetic scheme composed of four irreversible processes to reproduce SA/V-dependent bacterial growth curves; B = aerobic bacteria, B_an_ = anaerobic bacteria, and N = nutrient. The parameter expression *k*_2_/(*k*_4_[CO_2_] + 1) in step (III) represents the autoinhibition by CO_2_ where *k*_4_ is a control parameter to tune the inhibition strength. The reaction arrow in step (IV) symbolizes the physical escape of CO_2_ from the system.

Our chemical kinetics model (Scheme 1) [18] was extended to mimic flow conditions and the continuous input of the CO_2_ inhibitor. As a novelty, the numerical execution includes the possibility to adjust the kinetic parameters with the aim of fitting experimentally observed data, which is essential to perform meaningful predictions as well as comparisons between the growth kinetics of different bacteria.

The kinetic scheme consists of two coupled autocatalytic processes, one autoinhibitory step and the relief of inhibition, all together giving rise to considerable dynamic complexity and possible nonintuitive behavior. Step (I) denotes the autocatalytic growth of the aerobic bacteria (B) limited by O_2_. It accounts for the common “lag” and “exponential” phase. Process (II) stands for the conversion of aerobic to anaerobic bacteria (B_an_). Step (III) is the autocatalytic growth of anaerobic bacteria limited by the nutrient (N) and inhibited by CO_2_. It represents the SA/V-dependent and quasilinear growth stage. Step (IV) stands for the escape of CO_2_ from the system giving rise to a relief of inhibition and causing the dependence on the SA/V.

Since all processes are coupled, the kinetic scheme gathers together the different growth stages over the whole time-evolution into one single model. The corresponding set of reaction fluxes and differential equations are given in Table 1.

### 3.2. Initial Conditions and Parameter Estimation

The apparent 1 : 1 stoichiometry between B and O_2_ in step (I) of the kinetic scheme is hardly realistic with respect to a quantitative data treatment. It is more likely that many O_2_ molecules interact with one bacterium to cause an event such as the indicated self-replication. For that reason, we have used a normalized initial concentration of [B]_0_ = 4.0×10^−8^ M giving rise to a unity reaction order in [O_2_] of model step (I) (please see the Appendix for further details).

In the model, the escape rate of CO_2_ from the system was considered independent of the other components of the mixture. A semiquantitative reproduction of former measurements of the CO_2_ escape at different SA/V [17] delivers estimates for the values of the first-order rate constant *k*_3_ (CO_2_ → out) to be in the range between 10^−5^ and 10^−4^ s^−1^ for SA/V ratios between 0.14 and 2.5 cm^−1^.

### 3.3. Data Fitting of SA/V-Dependent* E. coli* Growth

The adjustment of the kinetic parameters to fit the experimentally observed growth curves was started manually with the “lag” and “exponential” growth stage and then extended to the entire kinetic curves. Subsequently, the automatic fitting procedures were carried out.

**Figure 2:**
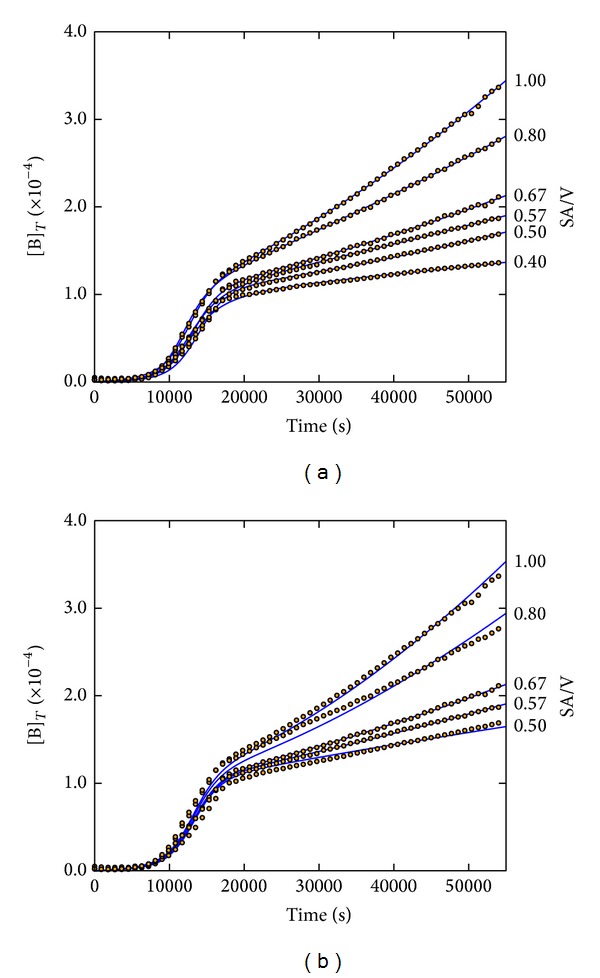
Series of EPEC growth experiments with SA = 1.0 cm^2^ and V = 1.0 to 2.5 cm^3^ corresponding to SA/V = 0.4 to 1.0 cm^−1^ (open circles) best fitted by the CKA model (solid lines). (a) Fitting of each growth curve independently with obtained values for *k*_0_ to *k*_4_ according to Table 2. (b) Only fitting of *k*_3_ at fixed *k*_0_ = 6.46 M^−1^s^−1^, *k*_1_ = 5.99 × 10^−5^ s^−1^, *k*_2_ = 2.18 × 10^−1^ M^−1^s^−1^, and *k*_4_ = 4.18 × 10^8^ M^−1^. Obtained values for *k*_3_ at different (SA/V): 1.29 × 10^−5^ (0.50), 6.18 × 10^−5^ (0.57), 1.07 × 10^−4^ (0.67), 2.97 × 10^−4^ (0.80), and 4.53 × 10^−4^ (1.00) s^−1^. Initial concentrations: [B] = 4.0 × 10^−8^, [O_2_] = 1.0 × 10^−4^, and [N] = 0.8 M, all others at zero.

Starting with the first attempts, we noticed a very good agreement (flexibility) between the model and the experimental data leading to excellent fitting results with respect to each individual experiment, with residual errors as small as 10^−12^ (Figure 2(a)). In these attempts, all kinetic parameters for the individual fitting of each of the kinetic curves were allowed to vary until the minimum residual error between simulated and experimental data was reached.  Table 2 shows the obtained parameter variations and the parameter mean values for the 6 fitted kinetic curves.

The data given in Table 2 indicates relatively small variations of the parameter values although each experiment was fitted independently. Note that the automatic fitting procedure has “chosen” parameter values for *k*_3_ that are in good agreement with the experimental data of the CO_2_ escape that we have measured for different SA/V ratios.

An exception to the small parameter variations is represented by the control parameter *k*_4_ that was implemented to tune the inhibition strength. In four experiments this parameter remained at a value of around 10^8^ M^−1^, but, in the experiments performed at SA/V = 0.8 cm^−1^, the order of magnitude was 6 and in the case of SA/V = 0.4 cm^−1^ it was 9. A less pronounced situation occurred for *k*_2_ in which the order of magnitude is usually −1 except at SA/V = 0.57 cm^−1^ where its value was −2 and at SA/V = 0.8 cm^−1^ where its value was −3. These variations could be the consequence of a parameter coupling between *k*_3_ and *k*_4_ and the further relationship between *k*_2_ and *k*_4_ in the rate expression *r*_III_.

Table 2 gives insight into the relative velocities of the assumed key processes for the SA/V-dependent EPEC growth. For instance, the autocatalytic growth of B_an_ (B_an_ + N →2B_an_ + CO_2_) is predicted to occur significantly slower than that of B (B + O_2_ → 2B), *k*_2_/*k*_0_ ≈ 0.02. Furthermore, the process B → B_an_ appears to be the slowest step in the model that apparently determines the overall rate.

Although the parameter variations were found to be rather small during the individual fitting procedures, a more challenging and realistic task for the CKA should be to keep the parameters *k*_0_, *k*_1_, *k*_2_, and *k*_4_ at fixed values for the 6 experiments and only to allow variations in *k*_3_ in order to reflect the differences in the SA/V ratios of the experimental series, thus assuming that all the other parameters are invariant regardless of changes in the scaling properties of the system.

The results of such attempt where exclusively *k*_3_ has been adjusted during the automatic fitting procedure and all the other parameters were kept at fixed values, that is, the same *k*_0_, *k*_1_, *k*_2_, and *k*_4_ for all 6 experiments, are shown in Figure 2(b). This data fitting was employed by (1) selecting arbitrarily one experiment (SA/V = 0.67 cm^−1^) to start with, (2) keeping *k*_3_ at a reasonable order of magnitude in accordance with the experimental observations of the CO_2_ escape, (3) performing the adjustment of *k*_0_, *k*_1_, *k*_2_, and *k*_4_, and then (4) using the adjusted parameters for SA/V = 0.67 cm^−1^ for the other experiments by exclusively adjusting *k*_3_.

As anticipated by the previous fitting results, we obtained an excellent fitting of the SA/V = 0.67 cm^−1^ growth curve. But also for the other experiments between SA/V = 0.5 and SA/V = 1.0 cm^−1^ a very reasonable data reproduction was obtained. In contrast, the fitting attempt of the SA/V = 0.4 cm^−1^ curve with the most horizontal SA/V-dependent growth phase failed. This could be due to experimental data scattering as well as hidden parameters or may reflect the need to include additional model steps. Nevertheless, the fitting of five individual experiments by only changing *k*_3_ can be considered as satisfactory.

**Figure 3:**
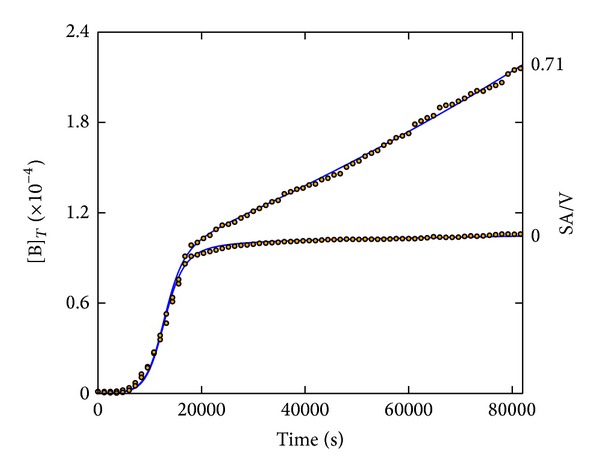
EPEC growth curves at SA/V = 0.71 and 0 cm^−1^ with V = 1.4 cm^3^ in both cases (open circles) best fitted by the CKA model (solid lines). Obtained parameter values: *k*_0_ = 7.87 M^−1^s^−1^, *k*_1_ = 1.98 × 10^−4^ s^−1^, *k*_2_ = 1.73 × 10^−3^ M^−1^s^−1^, *k*_3_(0.71) = 6.55 × 10^−3^ s^−1^, *k*_3_(0) = 0 s^−1^, and *k*_4_ = 4.35 × 10^8^ M^−1^.

Figure 3 shows the fitting of two experiments performed under significantly extended time and with a completely obstructed surface (SA/V = 0 cm^−1^) as well as an entirely free surface (SA/V = 0.71 cm^−1^) of the bacterial cultures by using culture volumes of 1.4 cm^3^ in both cases. Although the fitted parameters are not identical to those given in Figure 2, since dealing with two independent experimental series, they all lie very close to the observed parameter ranges of Table 2.

These results reveal that the model can respond properly to the two extreme culture surface conditions of “open” versus “closed” causing a marked difference in the later growth rates. Interestingly, the curve shape of the SA/V = 0 cm^−1^ case resembles the typical S-shaped portrays of bacterial growth kinetics [6], which was reproduced in the present case by keeping *k*_3_ at zero. Such situation also occurs when the culture surface is not completely obstructed but the SA/V becomes sufficiently small. In fact, this is the case for one standard* in vitro* culturing condition, that is, the growth in a static tube filled with culture medium. Hence, based on our observations, the later stage growth usually appears stationary (zero growth rate) but only due to a small SA/V, that is, because of the very slow escape of the inhibiting CO_2_.

### 3.4. Data Fitting of SA/V-Dependent* E. coli JM83*,* Salmonella typhimurium,* and* Vibrio cholerae* Growth

**Figure 4:**
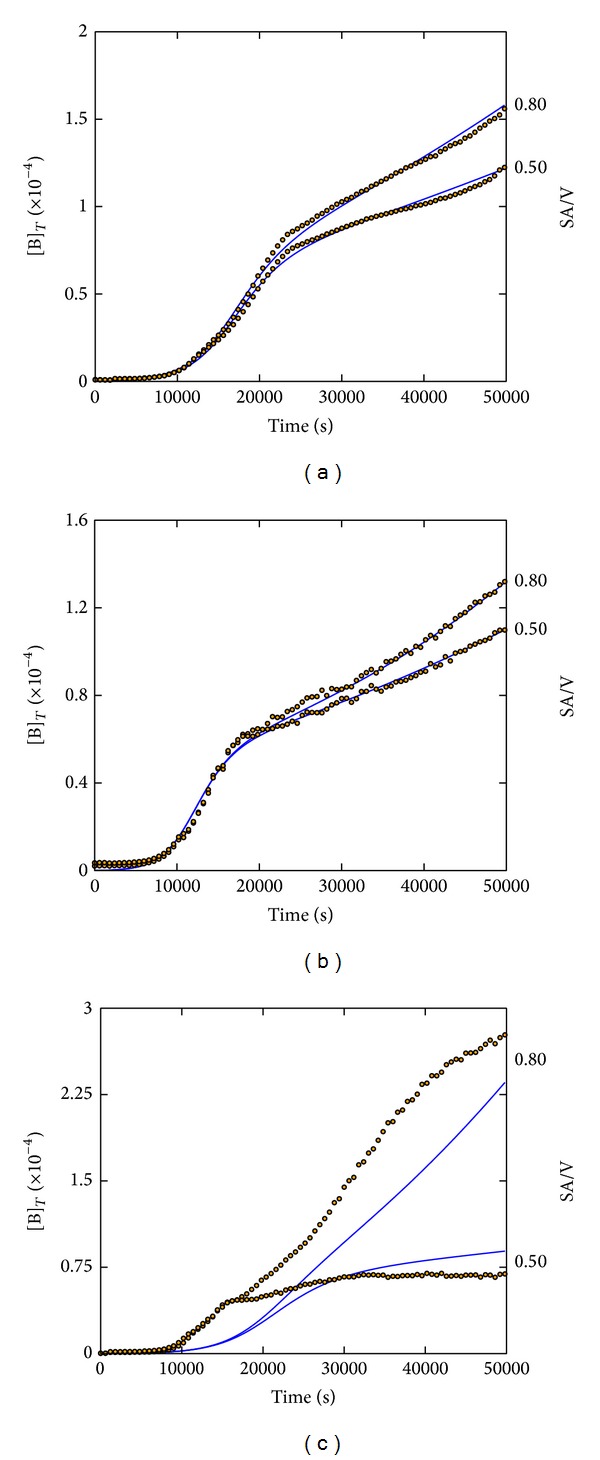
Experimental growth curves of various bacteria (open circles) best fitted by the CKA model (solid lines). (a)* E. coli JM83*: SA/V = 0.5 and 0.8 cm^−1^, obtained parameter values are given in Table 3. (b)* Salmonella typhimurium*: SA/V = 0.5 and 0.8 cm^−1^, obtained parameter values are given in Table 3. (c)* Vibrio cholerae*: SA/V = 0.4 and 1.0 cm^−1^, shown curves illustrate that a satisfactory data fitting was rejected. All initial concentrations for the simulations are given in Figure 2.

Our current experiments showed that the SA/V growth effect is not restricted to EPEC. In fact,* Vibrio cholerae*,* E. coli JM83,* and* Salmonella typhimurium*, at least, also respond to changes in the scaling properties during the later stage growth phases. However, a simple eye inspection of the shape of these growth curves shown in Figure 4 indicates differences with respect to the formerly recorded EPEC growth curves. Specifically, the later growth stage in the other bacterial species is curved while that of EPEC appears linear.

We subjected this experimental data to our modeling approach. Again, for the fitting of each bacterial system, only *k*_3_ was changed to account for the different SA/V. Since all systems were different also the extracted kinetic parameter values differ between EPEC,* Vibrio cholerae,* and* E. coli JM83*.  Table 3 provides an overview of the fitting results in comparison to the previous EPEC fitting. In general, the obtained values for* E. coli JM83* are closer to EPEC than those of* Salmonella typhimurium*.

As indicated in Figure 4, a reasonably good experimental data fitting could be obtained for* E. coli JM83* and* Salmonella typhimurium* while an acceptable fitting for* Vibrio cholerae* was impossible.

The fitting results of EPEC,* E. coli JM83*, and* Salmonella typhimurium* versus* Vibrio cholerae* are particularly interesting in two aspects:

1. from the viewpoint of bacterial phylogeny there is a difference between EPEC,* E. coli JM83,* and* Salmonella typhimurium* on one side and* Vibrio cholerae* on the other side [21]. We assume that these phylogenetic differences have an impact on the mechanism responsible for the growth kinetics. This implies that our model can discriminate between EPEC “like” and “unlike” growth dynamics as given by the circumstance that—based on a fixed model structure—in one case a reasonable set of parameter values can be obtained while in the case of* Vibrio cholerae* it cannot be obtained;
2. our model, despite its apparent flexibility, cannot fit any number of bacterial growth curves, indicating a certain specificity that, to our opinion, only the CKA can reveal. Such specificity was already observed during the model design, for instance, when we tested different degrees of autocatalysis that ended up as quadratic in the proposed model. The potential behavior of a cubic autocatalytic process in model step (I), 2B + O_2_ → 3B instead of B + O_2_ → 2B, resulted in the impossibility of fitting any of the experimental EPEC data, even for the individual fitting attempts.

Hence the model in its present structure not only proves valid because of its capacity to fit various experimentally observed data but also appears specific by apparently rejecting the fitting of EPEC “unlike” growth dynamics.

### 3.5. Prediction of Chemostat Behavior

**Figure 5:**
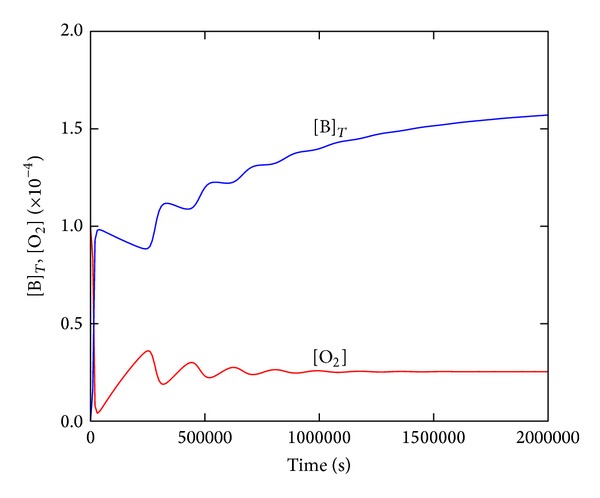
Simulation of EPEC growth behavior in an open-flow bacterial chemostat predicting an oscillatory approach to the steady states of the total bacteria and oxygen concentrations. The same parameters as in Figure 3 (SA/V = 0.71), flow rate constants *k*_5_ = *k*_6_ = 2.0 × 10^−6^ s^−1^; [B]_0_ = 4.0×10^−8^, [O_2_]_0_ = 1.0 × 10^−4^, and [N]_0_ = 8.0 × 10^−3 ^M, all others at zero.

We have evaluated the behavior of our model under hypothetical flow conditions with the continuous inflow of the reactants B and O_2_ and the corresponding outflow of the reaction mixture simulating a continuously fed and well-mixed bacterial chemostat [22]. The parameter values and initial conditions were taken from the fitting of the experiment SA/V = 0.71 cm^−1^ in Figure 3 as a prototypical case. As shown in Figure 5, some interesting transient behavior was observed before the concentrations reached stationary values. At specific flow rates, all concentrations are approaching their stationary states in a damped oscillatory manner. Note that this predicted behavior lasts very long.

Sustained oscillations or kinetic bistability by systematic variations of the flowrate was not observed or identified in the given parameter space. However, the simulations indicate complex dynamic behavior under flow conditions that could be of importance for practical purposes when EPEC-like organisms are grown in a bacterial chemostat. Moreover, the oscillatory approach to stationary bacterial concentrations could have an impact on any open bacterial growth system, such as the* in vivo* duplication of pathogenic bacteria and their expression of virulence.

The flow term extension of the model equations was also used to simulate the continuous input of the inhibitory CO_2_ into the semiopen batch system. As expected, a significant decrease in the rate of the later stage growth phase occurs. In contrast, an initial oversaturation of the cultures with CO_2_ under batch conditions is predicted to have no effect on the simulated bacterial growth curves due to the escape of CO_2_ before the SA/V-dependent growth phase was reached.

## 4. Conclusions

In this paper, we have applied the chemical kinetics approach (CKA) to rationalize and reproduce still unrecognized bacterial growth that was dependent on the liquid-surface-area-to-volume ratio (SA/V) of the bacterial cultures. Like in its previous applications, for instance, in the fitting of* Staphylococcus aureus* growth kinetics [3], the CKA was confirmed as a useful tool to understand the bacterial growth dynamics from a kinetic point of view as well as to suggest predictions of complex behavior under open-flow conditions.

As a novel feature, we found that the CKA combined with data fitting can serve to discriminate between different growth dynamics as well as to recognize similarities in growth mechanisms. This was demonstrated by the grouping of EPEC,* E coli JM83,* and* Salmonella typhimurium* on the one hand and* Vibrio cholerae* on the other hand. Furthermore, we have shown that the CKA can also apply to more complex growth dynamics with the possible extraction of rate parameter values or their relations that may be used to assess the relevance of the key processes that give rise to the curve shapes of the bacterial time-evolution.

It is important to underscore that the applied fitting procedures were entirely different from classical curve fitting because in the present case we performed an adjustment of the kinetic parameters of a fixed set of differential equations and did not use arbitrary equations as in usual curve fitting approach. The fitting proceeded by an automatic error minimization between the simulated and the experimental data. Usually, if a model is “wrong” no satisfactory parameter adjustment can be achieved. This case could apply to the* Vibrio cholerae* system. Hence a successful fitting, that is, a small residual error between experimental and simulated data like in the cases of EPEC,* E coli JM83,* and* Salmonella typhimurium*, indicates that the chosen model was a reasonable proposal.

Upcoming studies will be devoted to further experimental insight into the SA/V effect such as the examination of corresponding bacterial growth under open-flow conditions and a finer-grained model outline based on additional biochemical as well as kinetic information.

## Figures and Tables

**Table 1:** Kinetic setup of the CKA model. The parameters *k*_5  _ and *k*_6_ are flow rate constants to simulate chemostat conditions or the inflow of CO_2_ into the system. [B]_0_, [O_2_]_0_, [CO_2_]_0_, and [N]_0_ are the reactant inflow concentrations (B_an_ was not considered). The experimentally observed optical densities were taken as directly proportional to the total bacteria concentration [B]_*T*_ = [B] + [B_an_].

Reaction fluxes	Differential equations
*r*_I_ = *k*_0_ *·*[B]*·*[O_2_]	*d*[B]/*dt* = *r*_I_ − *r*_II_ + *k*_5_ *·* ([B]_0_−[B])
*r*_II_ = *k*_1_ *·*[B]	*d*[O_2_]/*dt* = −*r*_I_ + *k*_5_ *·* ([O_2_]_0_−[O_2_])
*r*_III_ = *k*_2_/(*k*_4_ *·*[CO_2_] + 1) *·* [B_an_] *·* [N]	*d*[B_an_]/*dt* = *r*_II_ + *r*_III_ − *k*_5_ *·* [B_an_]
*r*_IV_ = *k*_3_ *·*[CO_2_]	*d*[CO_2_]/*dt* = *r*_III_ − *r*_IV_ + *k*_6_ *·* ([CO_2_]_0_−[CO_2_])
	*d*[N]/*dt* = −*r*_III_ + *k*_5_ *·* ([N]_0_−[N])

**Table 2:** Minima, maxima, and arithmetic mean of the kinetic parameters after the individual fitting of the 6 kinetic curves shown in Figure 2 corresponding to SA/V ratios of 0.4, 0.5, 0.57, 0.67, 0.8, and 1.0 cm^−1^.

Parameter	Min	Max	Mean
*k*_0_ (M^−1^s^−1^)	5.97	9.96	7.77
*k*_1_ (s^−1^)	3.62 × 10^−5^	4.24 × 10^−4^	1.87 × 10^−4^
*k*_2_ (M^−1^s^−1^)	1.43 × 10^−3^	2.50 × 10^−1^	1.60 × 10^−1^
*k*_3_ (s^−1^)	2.41 × 10^−5^	2.41 × 10^−4^	1.02 × 10^−4^
*k*_4_ (M^−1^)	1.26 × 10^6^	1.08 × 10^9^	4.38 × 10^8^

**Table 3:** Obtained parameter values by fitting of EPEC, *E. coli JM83,* and *Salmonella typhimurium* growth curves at SA/V = 0.5 and 0.8 cm^−1^.

Parameter	EPEC	*E. coli JM83 *	*Salmonella typhimurium *
*k*_0_	6.46	9.91	16.11
*k*_1_	5.99 × 10^−5^	6.10 × 10^−4^	1.11 × 10^−3^
*k*_2_	2.18 × 10^−1^	3.91 × 10^−3^	3.08 × 10^−5^
*k*_3_ (0.5)	1.29 × 10^−5^	4.69 × 10^−3^	3.32 × 10^−4^
*k*_3_ (0.8)	2.97 × 10^−4^	1.07 × 10^−2^	5.10 × 10^−3^
*k*_4_	4.18 × 10^8^	5.28 × 10^8^	7.69 × 10^4^

**Table 4:** Relationship between number of bacteria, optical density readings, and the concentrations of B and O_2_.

Number of bacteria	Optical density	[B] (mol/L)	[O_2_] (mol/L)
1.5 × 10^8^	0	2.5 × 10^−16^	1.0 × 10^−4^
3.75 × 10^11^	0.5	6.25 × 10^−13^	~0
7.5 × 10^11^	1.0	1.25 × 10^−12^	~0

## Appendix

According to experimental observations [18], the initial concentration of dissolved O_2_ in the culture medium of a typical growth experiment drops to nearly zero when the “exponential growth phase” ends and the “quasilinear,” SA/V-dependent, growth stage starts. In terms of a 1 : 1 stoichiometry between B and O_2_, the bacterial concentration at this point of O_2_ depletion should be equal to the initial concentration of the limiting O_2_. We estimate the initial O_2_ media concentration roughly as 10^−4 ^M based on Henry's law [23]. Given that the OD value for the transitions between the “exponential” and the “quasilinear” stage for all experiments is OD ≈ 0.5, where [O]_2_ ≈ 0 M, and taking the available information given in Table 4, the ratio [O_2_]_0_/[B] = 1.0×10^−4^/6.25×10^−13^ = 1.6×10^8^ is associated with the apparent kinetic order in O_2_ of model step (I), that is, with the number of O_2_ molecules that are required to drive one bacterium to a change.

To avoid numerical difficulties by using a very small initial [B] and a very high kinetic order in O_2_, the initial concentration of B was normalized by using the concentration equivalence [6.25 × 10^−13^⇔1.0 × 10^−4^] between the bacteria and consumed O_2_ at OD = 0.5 extrapolated to [2.5 × 10^−16^⇔4.0 × 10^−8^] at OD = 0. Hence for the simulations the initial bacterial concentration was normalized to 4.0 × 10^−8 ^M giving rise to a unity reaction order in [O_2_] of model step (I).
